# Trigeminal Trophic Syndrome as an Unusual Cause of Chronic and Non-Healing Ala Nasi Ulcer: A Case Report

**DOI:** 10.29252/wjps.9.3.346

**Published:** 2020-09

**Authors:** Ali Mohammadi, Ali Khojasteh, Farzaneh Khojasteh

**Affiliations:** 1 Department of Plastic and Reconstructive Surgery, Shiraz University of Medical Sciences, Shiraz, Iran;; 2 School of Medicine, Shiraz University of Medical Sciences, Shiraz, Iran

**Keywords:** Trigeminal trophic syndrome, Ala nasi ulcer, Paraesthesia, Pruritus

## Abstract

Trigeminal trophic syndrome is an unusual cause of facial ulcers that affects the sensitive area of the trigeminal nerve. Trigeminal trophic syndrome (TTS) is an unusual condition characterized by anesthesia, paraesthesias and ala nasi ulceration, following peripheral or central damage to the trigeminal nerve. We reported a 27-year-old man who presented with a left ala nasi ulcer accompanied by pruritus and paraesthesia for two months and one month before admission, he was a case of car accident that was admitted in ICU due to diffuse axonal injury (DAI). An underlying infectious, malignant and vasculitic cause for the ulcer was excluded by a skin biopsy. So awareness of the predisposing factors and clinical presentations of this important disfiguring condition seems to be necessary to ensure prompt diagnosis and treatment.

## INTRODUCTION

Trigeminal trophic syndrome (TTS) is an unusual cause of facial ulcers that affects the sensitive area of the trigeminal nerve. Prompt diagnosis and institution of therapy is crucial in the management of these patients. Often, patients are subjected to extensive tests and investigations to exclude more common causes of ulceration like infections, cutaneous malignancies and vasculitis. For patients whose evaluations are unyielding, it is imperative to consider TTS to be an important differential diagnosis.^1^ We reported a patient who presented with typical features of TTS. 

## CASE REPORT

**Fig. 1:**
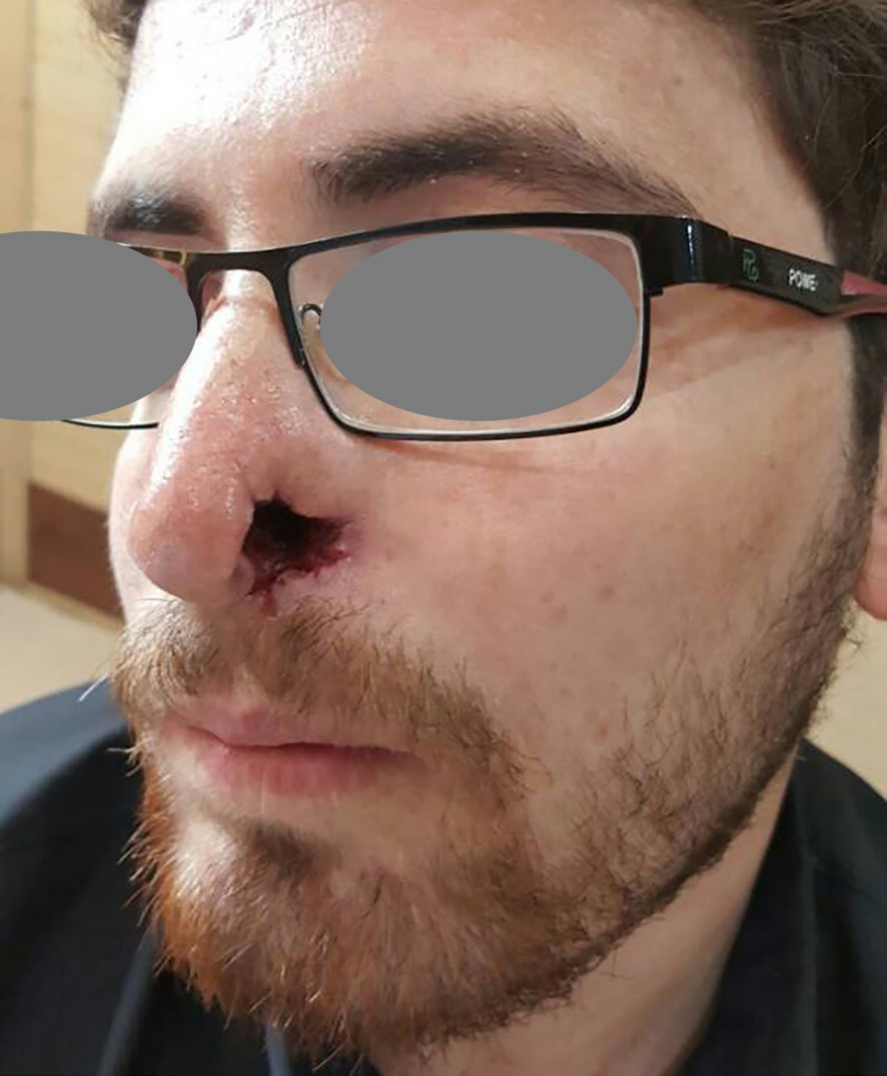
Clinical photograph shows the left ala nasi ulcer

A 27-year-old man with a left ala nasi ulcer accompanied by pruritus and paraesthesia for two months was presented and one month before admission, he was a case of car accident that was admitted in an intensive care unit (ICU) due to diffuse axonal injury (DAI) (Figure 1). He complained of “crawling” sensations and itching around the nose. A biopsy of the ulcer edge revealed a partially ulcerated lesion with no atypia along the adjacent intact epidermis. An underlying infectious, malignant and vasculitic cause for the ulcer was excluded by a skin biopsy. 

The patient was diagnosed with TTS based on the presence of facial paraesthesia, long history of repeated physical manipulation of the affected area, and the relevant history of head injury. He was educated about the self-induced nature of the ulceration and counseled to stop picking on the ulcer. All procedures performed were in accordance with the ethical standards of the institutional and national research committee and with the 1964 Helsinki declaration and its later amendments or comparable ethical standards. Informed consent was obtained from the patient.

## DISCUSSION

TTS is characterized by the appearance of one or more facial, strictly unilateral, ulcerations. Its characteristic location is the nasal wing, but it can also affect the frontal region, scalp, mouth, and other areas. TTS occurs after damage to the branches of the sensory nucleus of the trigeminal nerve.^1^ The tip of the nose is often spared because of a different innervation from the medial branch of the anterior ethmoidal nerve.^2^ This was also observed in our patient (Figure 1). TTS is a clinical diagnosis and should be suspected in all patients who present with facial ulceration and a relevant neurologic history, particularly if all the investigations and biopsies have been unyielding. TTS manifests as a chronic ulcer with minimal infiltrate without giant cells, granulomas or vasculitis on histological examination.^3^

In contrast to patients with factitial dermatitis, patients with TTS are often more ready to report paraesthesias and to be admitted for inflicting injuries on their skin. The latency period between the damage of the sensory nerve fibers and the appearance of the lesions is variable, ranging from weeks to decades.^2^ The pathogenesis underlying the sensation of itch after a cerebrovascular accident is not known. It may share the same mechanisms that possibly underlie intractable post-herpetic itching.^4^

TTS must be differentiated from other causes of facial ulceration, like basal or squamous cell carcinomas, pyoderma gangrenosum, Wegener’s granulomatosis, deep fungal infections, Mycobacterium tuberculosis infections, cutaneous leishmaniasis and sinonasal NK/T cell lymphoma which often involve the face.^5^^,^^6^ A good clinical history that includes exposure or travel history, symptoms of immunosuppression, history of neurological disease and a thorough systemic review is necessary to elucidate the correct diagnosis in these patients. Apart from a detailed physical examination, tissue cultures and skin biopsies are invaluable in the exclusion of other common causes of facial ulceration discussed earlier. Treatment mainly consists of patient education to prevent manipulation of the lesions and local measures. Gabapentin, carbamazepine, amitriptyline and alginate emulsions are some of the medical treatments used in TTS. Reconstructive surgery has also been undertaken for these patients.^7^

## CONCLUSION

TTS is a diagnosis of exclusion. Despite all these available measures, TTS remains a therapeutic challenge in most cases. In some cases, a multidisciplinary approach involving the neurological evaluation, psychological counseling for behavior modification, medical treatment and surgical repair is necessary.
